# Determining mutational burden and signature using RNA-seq from tumor-only samples

**DOI:** 10.1186/s12920-021-00898-y

**Published:** 2021-03-01

**Authors:** Erik Jessen, Yuanhang Liu, Jaime Davila, Jean-Pierre Kocher, Chen Wang

**Affiliations:** Mayo Clinic, 200 First Street SW, Rochester, MN 55905 USA

**Keywords:** Cancer, RNA-seq, UCEC, COAD, Mutational burden, Mutational signatures

## Abstract

**Background:**

Traditionally, mutational burden and mutational signatures have been assessed by tumor-normal pair DNA sequencing. The requirement of having both normal and tumor samples is not always feasible from a clinical perspective, and led us to investigate the efficacy of using RNA sequencing of only the tumor sample to determine the mutational burden and signatures, and subsequently molecular cause of the cancer. The potential advantages include reducing the cost of testing, and simultaneously providing information on the gene expression profile and gene fusions present in the tumor.

**Results:**

In this study, we devised supervised and unsupervised learning methods to determine mutational signatures from tumor RNA-seq data. As applications, we applied the methods to a training set of 587 TCGA uterine cancer RNA-seq samples, and examined in an independent testing set of 521 TCGA colorectal cancer RNA-seq samples. Both diseases are known associated with microsatellite instable high (MSI-H) and driver defects in DNA polymerase ɛ (POLɛ). From RNA-seq called variants, we found majority (> 95%) are likely germline variants, leading to C > T enriched germline variants (dbSNP) widely applicable in tumor and normal RNA-seq samples. We found significant associations between RNA-derived mutational burdens and MSI/POLɛ status, and insignificant relationship between RNA-seq total coverage and derived mutational burdens. Additionally we found that over 80% of variants could be explained by using the COSMIC mutational signature-5, -6 and -10, which are implicated in natural aging, MSI-H, and POLɛ, respectively. For classifying tumor type, within UCEC we achieved a recall of 0.56 and 0.78, and specificity of 0.66 and 0.99 for MSI and POLɛ respectively. By applying learnt RNA signatures from UCEC to COAD, we were able to improve our classification of both MSI and POLɛ.

**Conclusions:**

Taken together, our work provides a novel method to detect RNA-seq derived mutational signatures with effective procedures to remove likely germline variants. It can leads to accurate classification of underlying driving mechanisms of DNA damage deficiency.

## Background

### Mutational identity of cancers

Clinical treatment of cancer is largely dependent on the mechanistic defect(s) giving rise to a tumor; subsequently the discovery of biomarkers for identifying these defects has become increasingly important. PD-L1 inhibitors are immunotherapy drugs and function by repressing the ability of tumors to disguise themselves from immune system. PD-1 on T cells binds to the PD-L1 receptor on some types of tumor cells, signaling to the T cell not to target the tumor cell [1, 2]. Tumors that display high levels of genetic diversity are often susceptible to PD-L1 inhibitors as variants in the DNA sequences lead to the display of abnormal proteins on the tumor cell surface, allowing the immune system to attack the tumor in the absence of the PD-1/PD-L1 “off switch” [3]. Classification of patients as promising or unpromising candidates for PD-L1 inhibitors is therefore of great importance in therapeutic decisions.

Tumor Mutational burden (TMB) is a metric used to quantify the degree of mutational diversity in tumor cells by calculating the number of somatic variants per mega base. Different tumor types display highly different levels of mutational burden and high TMB is associated with response to immunotherapies [4].

Mutations in the catalytic subunit of DNA polymerase epsilon (POLɛ), responsible for DNA repair during chromosomal replication, have been associated with uterine and colorectal cancers with a large mutational burden [5, 6]. The mismatch repair system (MMR) repairs spontaneous mismatches in the DNA, and disruption of MMR leads to polymorphism in the length of microsatellite regions (microsatellite instability high, MSI-H, or microsatellite stable, MSS) [7]. Additionally, an increased mutational burden has been associated with MSI-H tumors [8].

Disruption of DNA repair mechanisms also results in a unique mutational signature as certain base substitutions occur more frequently based on the mechanism of repair, and with specific, adjacent sequence context. Mutational signatures are calculated over trinucleotide sequence context (e.g. ACA to AGA) resulting in 96 quantitative variables to compare as opposed to the single variable of mutational burden. The Catalogue of Somatic Mutations in Cancer (COSMIC) is a database of 30 mutational signatures identified in a variety of tumor types, including the signature of natural accumulation of mutations during aging.

### Current method for characterization of tumors

Classically [9], MMR status has been determined using PCR based methods, looking at five microsatellite regions for polymorphic changes in length, however these tests are effort, cost, and time intensive. More importantly there is a wide variation in mutational burden across MSI-H cases; hence there is a need for a global assay [10]. More recently, mutational signature models have been used to predict MMR and POLɛ status from variants identified from next generation sequencing [11]. However, routine practice requires both a tumor and normal sample, to allow for private, germline variants to be subtracted out. Additionally, it is common to use variants identified from DNA sequencing, which contains variants from the entire exome or genome, including variants not expressed. Expressed variants detected in RNA-seq are more likely to result in neo-epitopes, which are likely to be targeted by immunotherapy.

In this study, we investigated the efficacy of MSI-H and POLɛ classification from RNA -sequencing, using only a tumor sample without its corresponding normal sample. By using RNA sequencing we also get gene expression and gene fusion data for the tumor, and the variants we identify are expressed. Finally, only using the tumor removes the requirement to take a normal sample, but also provides the greatest challenge in requiring us to contend with the presence of germline variants, that can make up well over 99% percent of called variants. We tested both supervised and unsupervised approaches to identify the mutational signature of samples and created a regression based model. Based on identified signatures and associated coefficients, we demonstrated potentially clinical meaningful application of classifying MSI-H and POLE tumors, which are candidates for immunotherapies.

## Aim of study

Determine the efficacy of classifying MSI-H and POLɛ status from mutational signatures derived from tumor-only, RNA-seq data.

## Methods

### Materials

All raw UCEC and COAD RNA-seq data was acquired from The Cancer Genome Atlas (TCGA https://portal.gdc.cancer.gov/).

### Calling variants from RNA-seq TCGA data

Bam files downloaded from TCGA had variants called using RVboost [12]. RVboost provides a Q-score as a metric for confidence in the metric using the subsequent filtering steps.

### Filtering to enrich for somatic variants

Filtering to enrich for somatic variants was performed in three steps. Step 1 was to reduce the number of sequencing errors and required variants to have a Q-score > 0.05 and at least 25 supporting reads for the alternative allele. Step 2 removed common population variants by only accepting variants that were not present in the dbSNP database. Step 3 was to remove variants that had allele frequencies close to perfectly heterozygous (0.5) or homozygous (1). Any variants with an allele frequency between 0.45 and 0.55, or 0.95 and 1 were removed.

### Mutational burden

Mutational burden was calculated as the number of variants remaining after filtering divided by the number of sequenced mega bases with at least 50 read depth. Read depth was calculated using GATK DepthOfCoverage to find the number of bases at 50+ read depth.

### Regression of mutational signatures

Regression of the 96 vector mutational signatures to the COSMIC signatures (https://cancer.sanger.ac.uk/cosmic/signatures_v2) was performed in R.

### Unsupervised signatures

Unsupervised signatures were determined using the pre-existing R package mutationalPatterns (https://bioconductor.org/packages/release/bioc/html/MutationalPatterns.html).

### Validation

Validation was performed by taking random samplings of UCEC or COAD classifications and calculating recall and specificity. ROC plots were generated from classifications of the full dataset. Significance between two groups of data was determined by t-test.

To determine the percentage of true somatic variants identified through the enrichment steps (see Filtering to enrich for somatic variants), the enriched pool of variants from RNA-seq were compared to the identified somatic mutations from DNA-seq. DNA-seq somatic mutations were downloaded from TCGA.

### Identification of RNA editing events

A comprehensive list of RNA editing events was downloaded from REDIportal [13]. Variants from RNA-seq were compared to the list of RNA editing events to determine the fraction of RNA editing events per sample.

## Results

**Fig. 1:**
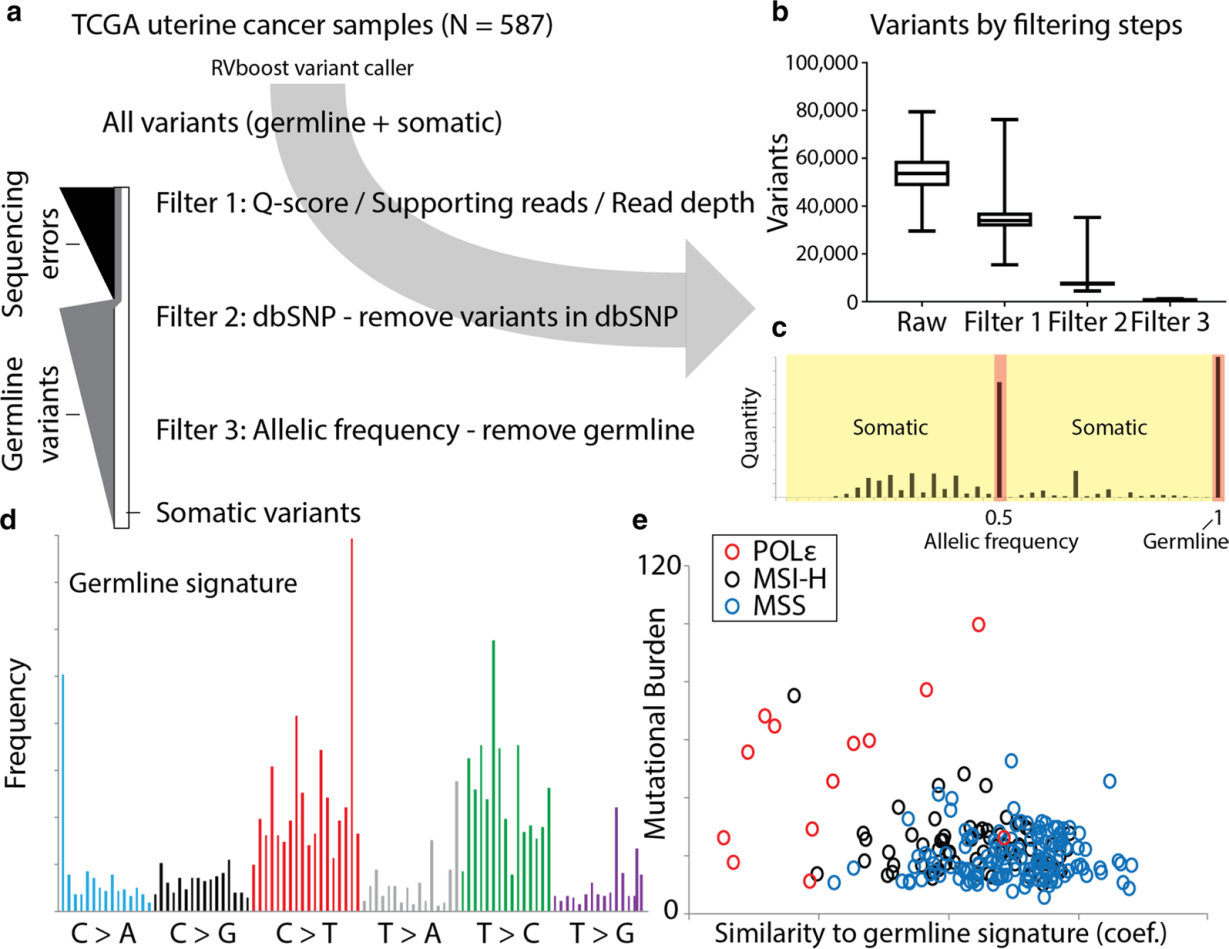
Filtering process to enrich for somatic variants in tumor-only, RNA-seq TCGA samples. **a** Filtering workflow to enrich for somatic variants. Step 1: filter based on Q-score and number of supporting reads to reduce sequencing errors appearing as variants. Step 2: remove all variants that are present in the population variant database dbSNP. Step 3: remove variants with allelic frequencies between 0.45 and 0.55, and 0.95 and 1. **b** Number of variants at each stage of the filtering process. **c** Allelic frequency plot for a representative sample with sub-clonal, mosaic variants in yellow and potential germline variants in red. **d** Mutational signature of germline variants derived from the variants filtered out by referencing dbSNP. **e** Mutational burden for all samples (MSS in blue, MSI-H in black, and POLɛ in red) compared to the regression coefficient to the germline (dbSNP) signature

To identify somatic variants from uterine corpus endometrial carcinomas (UCEC) and colon adenocarcinomas (COAD), RNA-sequencing alignment files from 587 UCEC and 521 COAD tumor samples were downloaded from The Cancer Genome Atlas (TCGA) and single nucleotide variants were called using the human reference hg38 genome (RVBoost, see methods) [14]. The median sample had approximately 55,000 called variants, a majority of which are expected to be private, germline variants. We implemented a series of filtering steps aimed at selectively removing germline variants and enrich for somatic variants (Fig. 1a).

The first step was to select for high confidence variants, removing false positives such as sequencing errors, by requiring a read depth of at least 50 reads at the position, with at least 20 reads supporting the alternative allele. This and subsequent filtering steps greatly reduced the number of variants from the original pool (Fig. 1b). The second step was to remove all population variants that have been previously characterized in the dbSNP database, as these variants would highly likely to be germline. The third step was to remove variants that had allelic frequencies between 0.45 and 0.55 (heterozygous) or 0.95 and 1 (homozygous). Tumor samples are often not 100% pure tumor, and additionally, tumors are often mosaic as mutations accumulate in subclones. As a result, the allelic frequencies of somatic variants deviate from germline variants of 0.5 and 1 (Fig. 1c). The number of variants in the resulting list enriched for somatic variants (referred to as the somatic variants for the remainder of the paper) was reduced by a median of 100-fold from the starting variant pool.

The mutational signature of germline variants was determined using the removed variants from filtering steps 2 and 3 (Fig. 1d), and used to estimate the remaining germline population in the final pool of enriched somatic variants. The mutational signature for each sample’s somatic variants was determined in a similar method to the COSMIC signatures and germline signature mentioned above. Linear regression of the trinucleotide frequencies for the somatic variant signatures against those of the germline signature gave a coefficient representing similarity to the germline (see methods). To determine if mutational burden, the number of somatic variants per sequenced Mb, was a function of variability in somatic variant numbers or variability in the filtering process, resulting in left over germline variants, we tried to correlate mutational burden with the similarity to the germline signature (Fig. 1e). Mutational burden was independent of the similarity to the germline signature for all three cancer subtypes, POLɛ, MSI-H, and MSS, suggesting higher mutational burden isn’t due to contaminating germline signatures.

### Calling tumor mutational subtype using regression

**Fig. 2:**
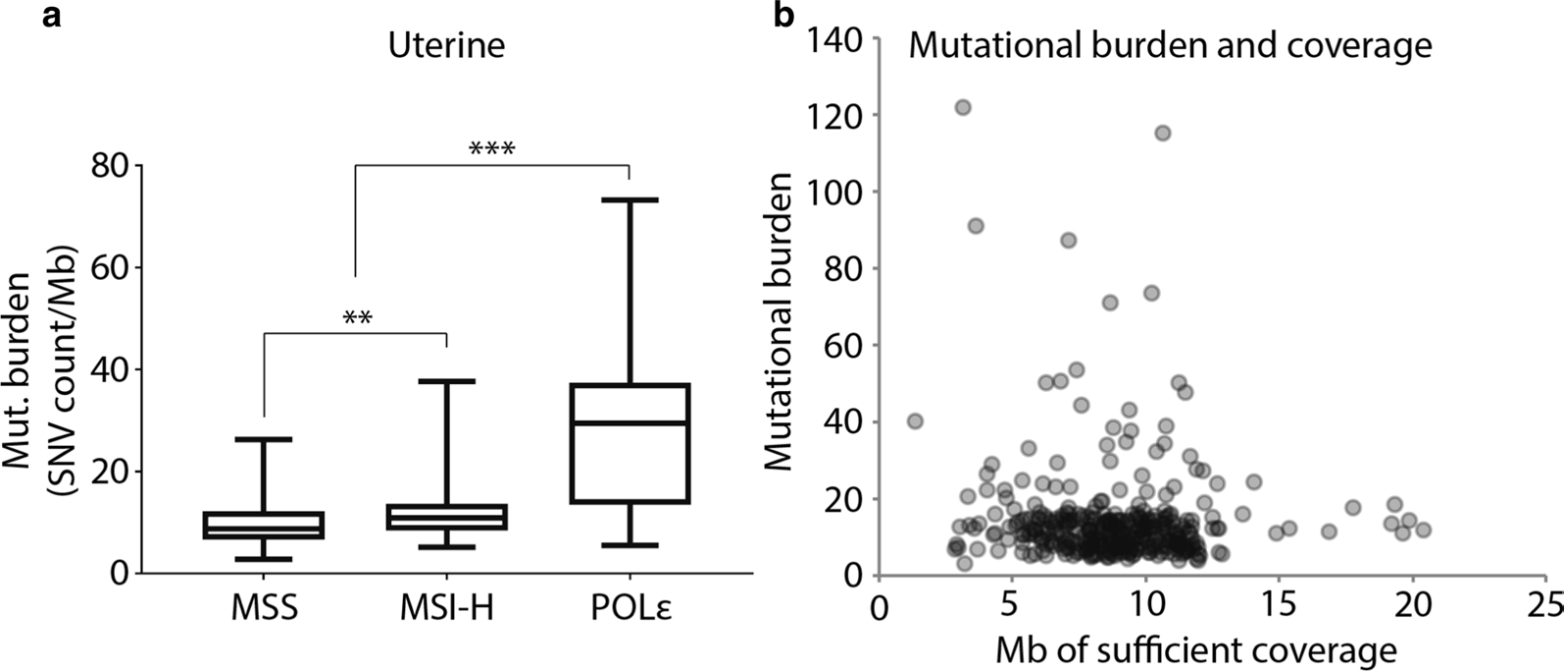
Mutational burden of filtered UCEC samples. **a** Mutational burden calculated from variants remaining after filtering to reduce the presence of germline variants. Significance from t-test had a p-value of < 0.01 for the MSS and MSI-H comparison, and < 0.001 for POLɛ against either MSS or MSI-H. **b** Mutational burden plotted against the number of mega bases of sequencing for each sample

We next determined if the mutational burden for the POLɛ and MSI-H tumor subtypes, both defined by disruption of DNA integrity mechanisms, was increased relative to MSS tumors, which should only acquire somatic variants from the natural aging process. The mutational burden of POLɛ samples showed significant separation from both MSS and MSI-H tumors (Fig. 2a, p-value < 0.001, unmatched t-test). MSI-H samples also showed statistically significant separation (p-value < 0.01, unmatched t-test), however there was substantial overlap between the mutational burden distributions of MSS and MSI-H samples. One concern was that samples sequenced at higher depth would show an increase in TMB, providing a confounding factor to analyzing tumor-only mutational burden. However, comparing the number of megabases with sufficient (> 25 read depth required by our variant filtering) for each sample to TMB showed no significant correlation (Fig. 2b, r = 0.015).

### Classification of MSS, MSI-H, and POLɛ samples based on supervised approaches

**Fig. 3:**
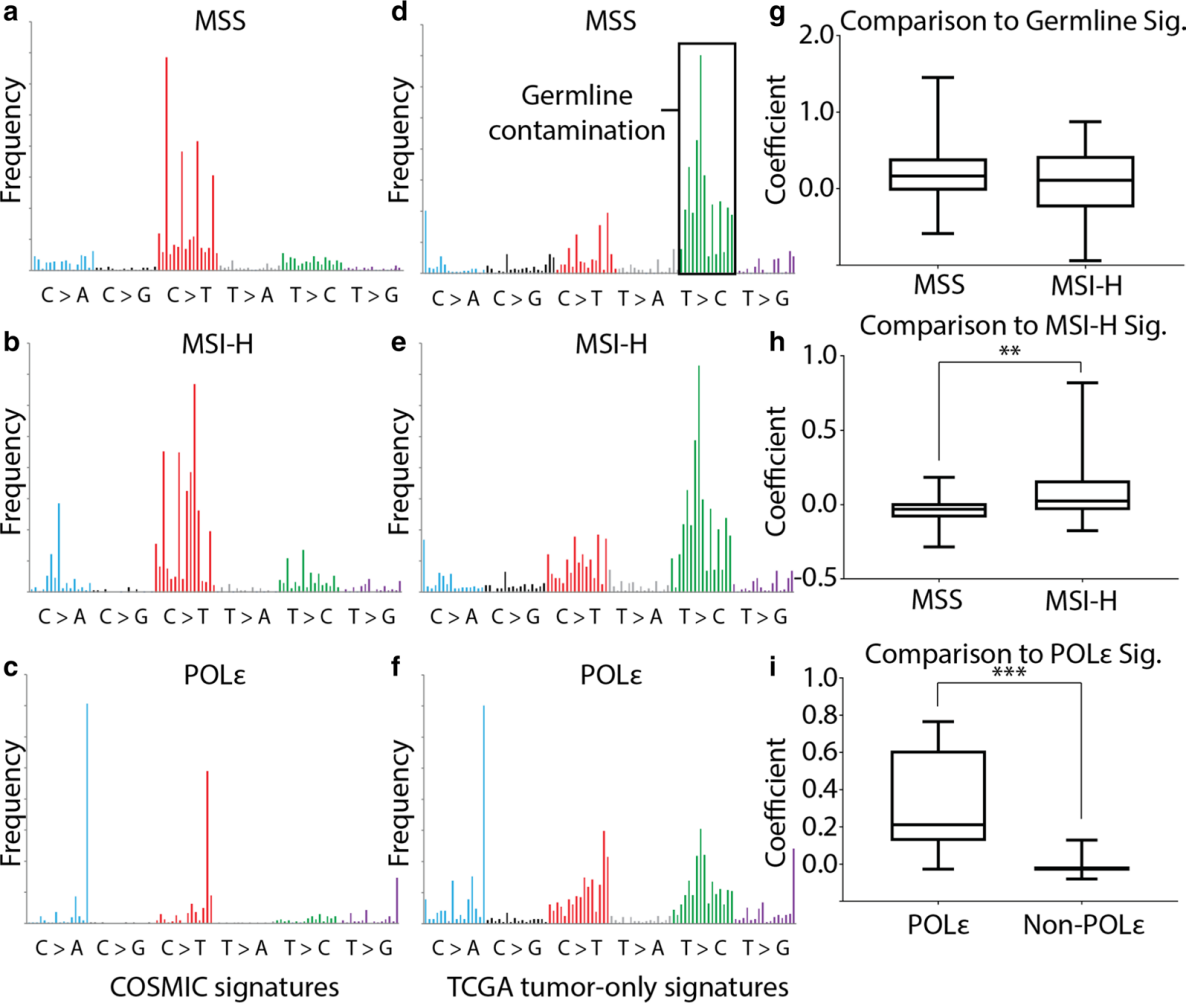
Mutational signatures from filtered UCEC variants. **a**–**c** COSMIC mutational signatures of MSS (natural aging, signature 5), MSI-H (signature 6), and POLɛ (signature 10). **d**–**f** Filtered UCEC variant derived signatures for MSS, MSI-H, and POLɛ. **g** MSS and MSI-H samples regressed against the germline signature derived from dbSNP variants. **h** MSS and MSI-H samples regressed against COSMIC MSI-H signature. Significance from t-test, p-value < 0.01. POLɛ and non-POLɛ samples regressed against COSMIC POLɛ signature. Significance from t-test, p-value < 0.001)

The mutational signatures for MSS, MSI, and POLɛ tumors from COSMIC (Fig. 3a–c), and the germline signature derived from dbSNP variants were compared to tumor-only TCGA samples. TCGA samples with clinical information on MSI and POLɛ status were grouped to make corresponding MSS, MSI-H, and POLɛ mutational signatures from the somatic variants passing through the filtering criteria (Fig. 3d–f). Increased frequency of T to C (green) changes suggests additional germline variants remain after filtering, influencing the signatures of MSS and MSI-H more than POLɛ.

**Fig. 4:**
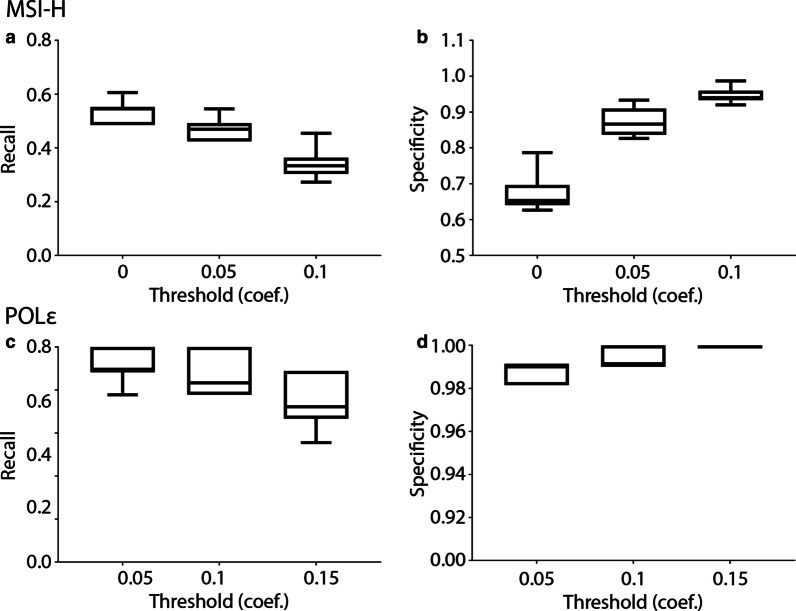
Performance of signatures from filtered UCEC variants against COSMIC signatures. Randomized testing groups were generated ten times to evaluate performance of the filtering process on the ability to classify tumor type. **a** The recall of MSI-H samples at different regression coefficient thresholds. **b** The specificity for MSI-H samples. **c** The recall of POLɛ samples. **d** The specificity for POLɛ samples

Regression of MSS and MSI-H samples against the germline signature showed no significant differences in the regression coefficients between the two groups (Fig. 3g). To test if POLɛ and MSI-H samples could be differentiated from MSS cases, we regressed each samples’ signature against MSI-H and POLɛ COSMIC signatures. MSI-H samples had higher regression coefficients than MSS samples against the MSI-H signature, although the overlap in the coefficient distribution is high, the MSI-H distribution is skewed toward higher correlations (Fig. 3h). POLɛ samples showed a stronger separation of coefficient values from non-POLɛ samples, likely due to the POLɛ signature being less disrupted by contaminating germline variants and distinct from either MSS or MSI-H signatures (Fig. 3i). Next, we tested the performance of the MSI-H and POLɛ signatures derived from the tumor-only TCGA samples against COSMIC signatures. Training and testing sets were randomly selected 10 times and used to calculate the recall and specificity. The POLɛ signature had strong recall and specificity, while MSI-H was moderate, consistant with our previous results (Fig. 4).

To quantify the per signature bias during regression to RNA samples, we ran 8000 normal GTEx samples through the pipeline. We found that background bias for each signature was relatively distinct and consistent (Additional file 1: Figure 1). Germline showed the highest coefficients, consistent with the idea that a majority of the GTEx variants should be germline, familial variants. The distributions of background coefficients can be subsequently used to derive statistics on how far samples are outliers and are likely to be a particular subtype.

### Identification of mutational signatures using an unsupervised approach

To expand the capabilities of the pipeline to potentially identify rare tumor subtypes we added the mutationalPatterns R package from bioconductor, a previously described, unsupervised approach [12]. Mutational signatures identified by mutationalPatterns looked comparable to the cosmic signatures used in the supervised approach (Additional file 1: Figure 2). Identified signature 3 corresponds to MSS samples, cosmic signature 5; identified signature 1 corresponds to MSI-H, cosmic signature 6; and identified signature 5 corresponds to POLɛ, cosmic signature 10. A majority of samples were explained by the five major signatures identified, and most showing strong correlation to only one signature (Additional file 1: Figure 3).

### Characterizing enriched somatic variants

We quantified the fraction of true somatic events in our population of enriched somatic variants from RNA-seq by comparing to the patient’s corresponding TCGA somatic variant list that was identified from a tumor-normal DNA-seq pair. The fraction of true somatic events in our RNA-seq variant lists correlated positively with the total number of somatic variants identified from DNA-seq (Additional file 1: Figure 4A).

Another complication with using RNA-seq is the presence of RNA editing events. To calculate the effect of RNA editing in our dataset, we downloaded a comprehensive list of known RNA editing events. The fraction of our enriched variants as the result of RNA editing was low, with 90% of samples having less than 1%, and 100% of samples having less than 4%, of variants identified in the RNA editing database (Additional file 1: Figure 4B).

### Validation using colorectal TCGA samples

**Fig. 5:**
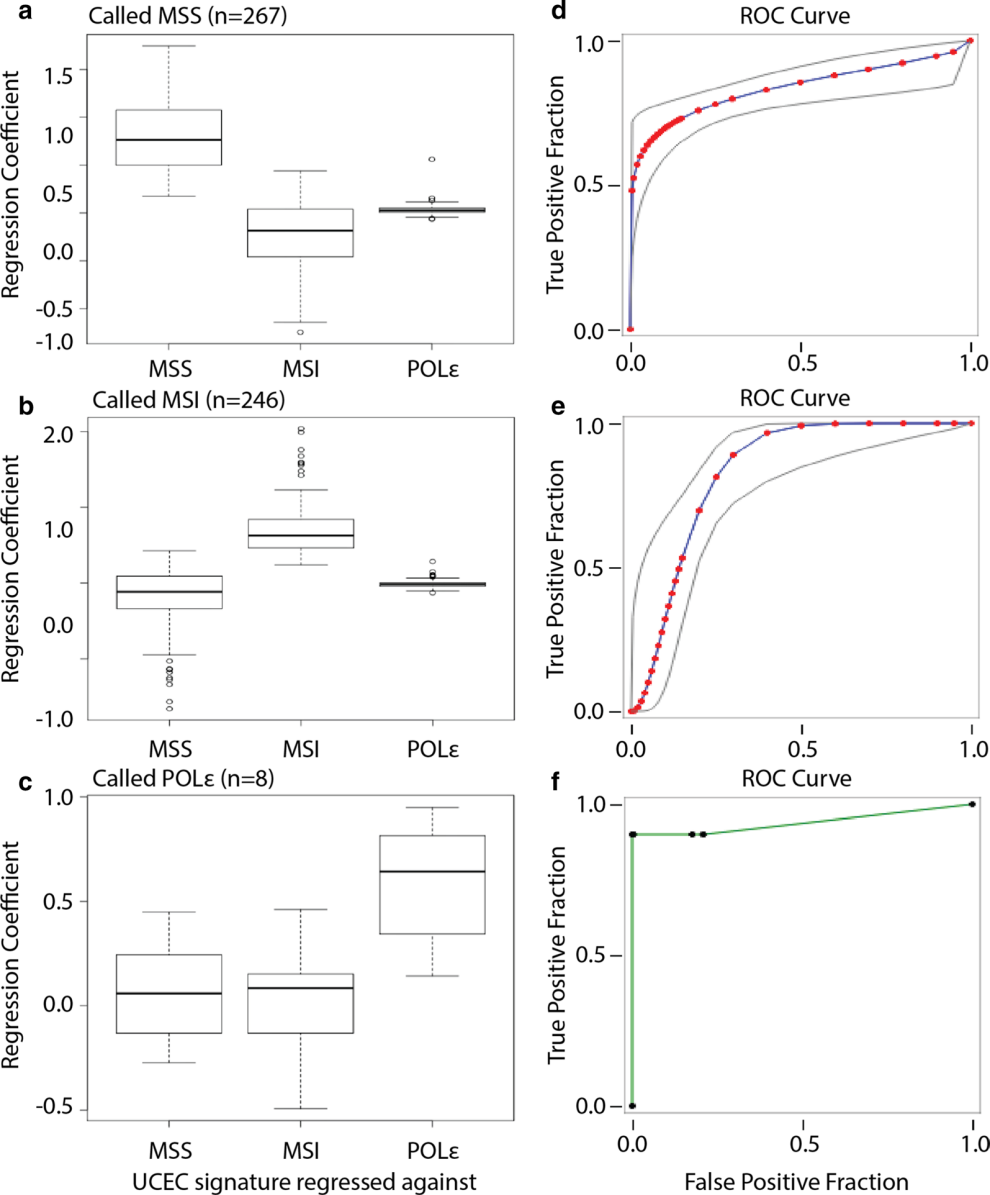
Performance of mutational signatures derived from UCEC tumor-only, RNA-seq on COAD samples. **a**–**c** Boxplots of COAD sample regression coefficients against MSS (natural aging), MSI-H, and POLɛ signatures derived from UCEC samples. **d**–**f** ROC plots of MSS, MSI-H, and POLɛ classification using the regression coefficients from signatures derived from UCEC samples

We next wanted to test the performance with signatures generated from the uterine data and applied to a similar dataset of colorectal (CRC) samples from TCGA. The mutational frequencies for each trinucleotide change of MSS, MSI-H, and POLɛ uterine samples were used to generate a de-novo signature from the tumor-only, mRNA sequencing data. Generated signatures were then regressed against the mutational frequencies for 521 colorectal tumors, of which 208, had MSS, MSI-H, and POLɛ subtype annotated. The regression coefficients for samples of each subtype were elevated for comparisons against the corresponding de-novo signatures (Fig. 5a–c). The de-novo signatures were able to distinguish between MSS and MSI-H more effectively, and POLɛ just as effectively, compared to regression against the COSMIC signatures (Fig. 5d–f). Taken together, tumor subtype is able to be effectively called when using signatures accounting for the method specific biases encountered from using the RNA of samples lacking a paired normal.

## Discussion

The difficulty in reducing the ratio between germline and somatic variants for the purpose of determining tumor subtype from mutational signature initially resulted in MSS and MSI-H subtypes being nearly indistinguishable. Filtering of variants using quality scores, databases, and allelic frequency greatly reduced the number of germline variants, but there was still significant germline contamination in the mutational signatures of subtypes with low tumor mutation burden. Additionally, the similarity between MSS and MSI-H signatures further convoluted distinguishing between the two subtypes. The increased mutation burden of POLɛ tumors, resulting in a higher ratio of somatic to contaminating germline, and the mutational signature being distinct from germline, MSS, or MSI-H signatures combined to allow for POLɛ status to be called from the mutational signature.

Further improving the method by using uterine samples to generate mutational signatures for MSS, MSI-H, and POLɛ subtypes increased the ability to distinguish between MSS and MSI-H tumors in colorectal samples, while maintaining the performance of POLɛ tumors. The improvement coming from using the generated signatures may be the result of intrinsically modeling in germline contamination and biases specific to mRNA sequencing. Future directions could include clustering MSS or MSI-H signatures and regressing against sub-signatures within the subtypes, which may become increasingly important as the number of tumor types analyzed increases.

The ability to call MSS, MSI-H, and POLɛ status from tumor-only samples would simplify the testing process. Often paired normal samples are not taken with the tumor, or are normal tissue adjacent to the tumor and may confound results by contain some percentage of tumor cells or lack the amount of sample to generate quality data. Although we acknowledge the tradeoff of being unable to fully remove germline variant contamination from the tumor mutational burden and signatures, we believe being able to classify tumors from one standalone sample, and the cost and logistical benefits that accompany that, are worth continued development. Additionally, the effect of complicating factors such as RNA editing seem to negligible.

Another novel advantage our approach has is the use of RNA sequencing data to identify the variants used to calculate mutational burden and generate the signature. The concept of tumor mutational burden is that more mutations in the DNA will lead to abnormal proteins being expressed on the cell surface, which would allow the patient’s immune system to distinguish the tumor as non-self. Variants from RNA sequencing are only those that are expressed from genes, giving a more biologically relevant calculation of tumor mutational burden. Additionally, RNA sequencing gives information on gene expression, alternative splicing, and gene fusions that is not present from DNA based methods. Altogether, our approach provides another facet of information from an already useful technique.

Correct identification of tumor subtype as MSS, MSI-H, or POLɛ can lead to important patient treatment decisions, improving the quality of care offered. As medicine moves towards a more individualized approach, a higher focus is placed on characterizing the tumor and personalizing the treatment. POLɛ tumors are such a case, as previously mentioned, a higher tumor mutational burden leads to tumor cells presenting abnormal proteins. These tumors often compensate by over expressing PD-L1 to disguise the tumor from the immune system. Subsequently, these tumors are good candidates for combination treatment with PD-L1 inhibitors, allowing the immune system to target the tumor in addition to other treatments. Developing easier and more accessible methods to properly classify tumors are therefore important to the advancement of patient care.

## Conclusions

Mutational burden and signatures of POLɛ are easily identifiable from RNA-seq, tumor only samples. We can call POLɛ samples using mutational burden and/or mutational signatures using linear regression or unassisted learning.

## Supplementary Information

**Additional file 1**: **Fig S1**: Background regression coefficients of COSMIC signatures in UCEC samples. Histogram of UCEC regression coefficients against (A) a germline signature derived from dbSNP variants, (B) natural aging signature (COSMIC signature 5), (C) MSI-H signature (COSMIC signature 6), and (D) POLɛ signature (COSMIC signature 10). **Figure S2**: Unsupervised mutational signatures. The mutational frequencies of all five unsupervised signatures identified from UCEC samples and output by mutationalPatterns R package. **Figure S3**: Sample correlations to unsupervised identified signatures. Sample mutational frequencies were correlated to the top five signatures output by the unsupervised method. The samples are color coded (y-axis bars) based on the clinical annotation of the tumor in regares to MSI and POLɛ status.

## Data Availability

The tool and an example dataset of 100 annotated vcfs are available at the following Github repository: https://github.com/Jessen-Erik/RNA.TOMS. Raw RNA-seq data is available on TCGA.

## Abbreviations

TCGA: The Cancer Genome Atlas
UCEC: Uterine Corpus Endometrial Carcinoma
COAD: Colon Adenocarcinoma
TMB: Tumor Mutational Burden
COSMIC: Catalogue Of Somatic Mutations In Cancer
MMR: Mismatch Repair
MSI-H: Microsatellite Instability High
POL: DNA Polymerase Epsilon
