# Supplementary figures and images for: Determining mutational burden and signature using RNA-seq from tumor-only samples
Source: BMC Med Genomics. 2021 Mar 1;14:65. doi: 10.1186/s12920-021-00898-y (PMC7923324; doi:10.1186/s12920-021-00898-y)

Supplementary Figure 1:

**
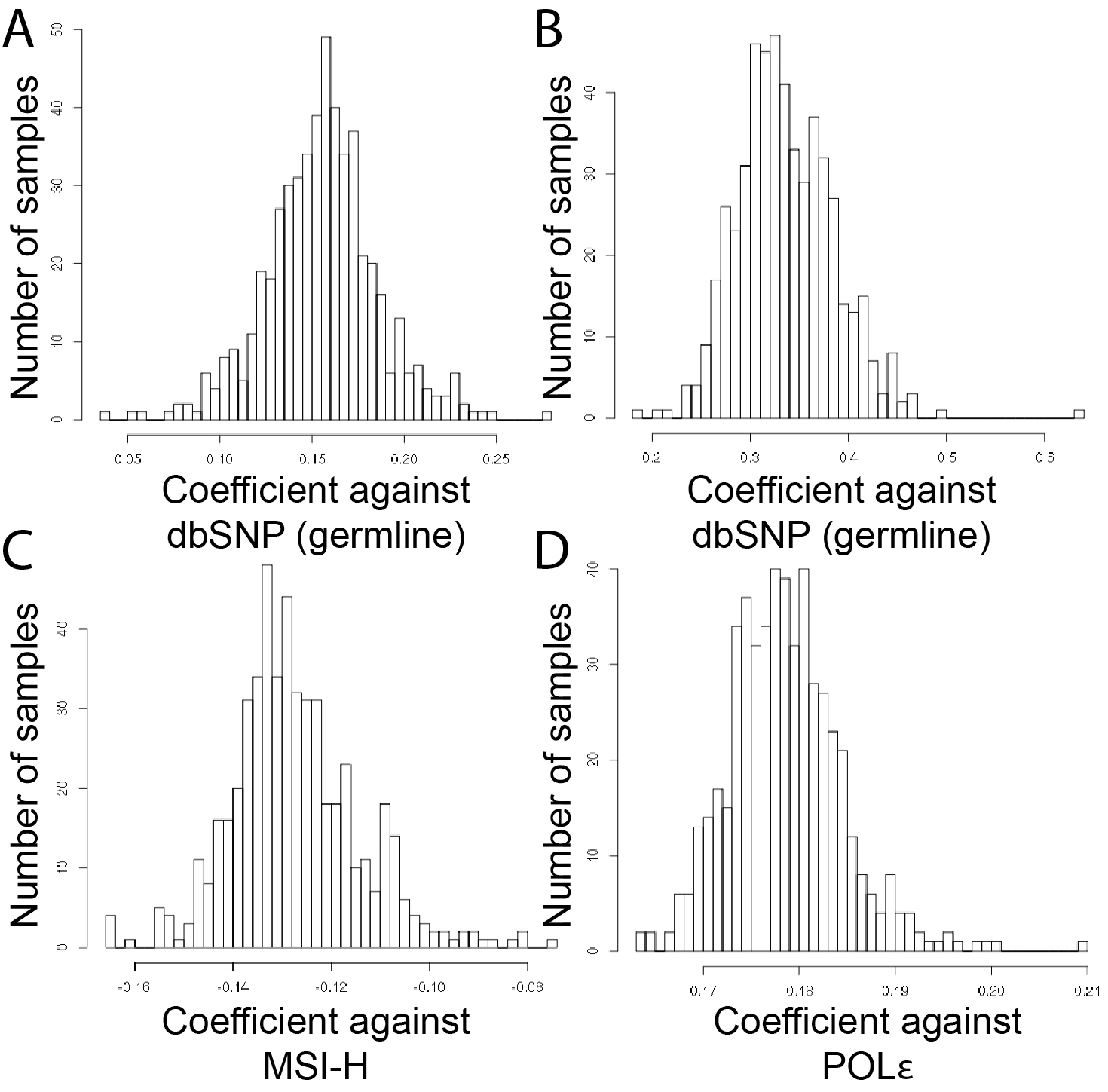
**

Supplementary Figure 2:

**
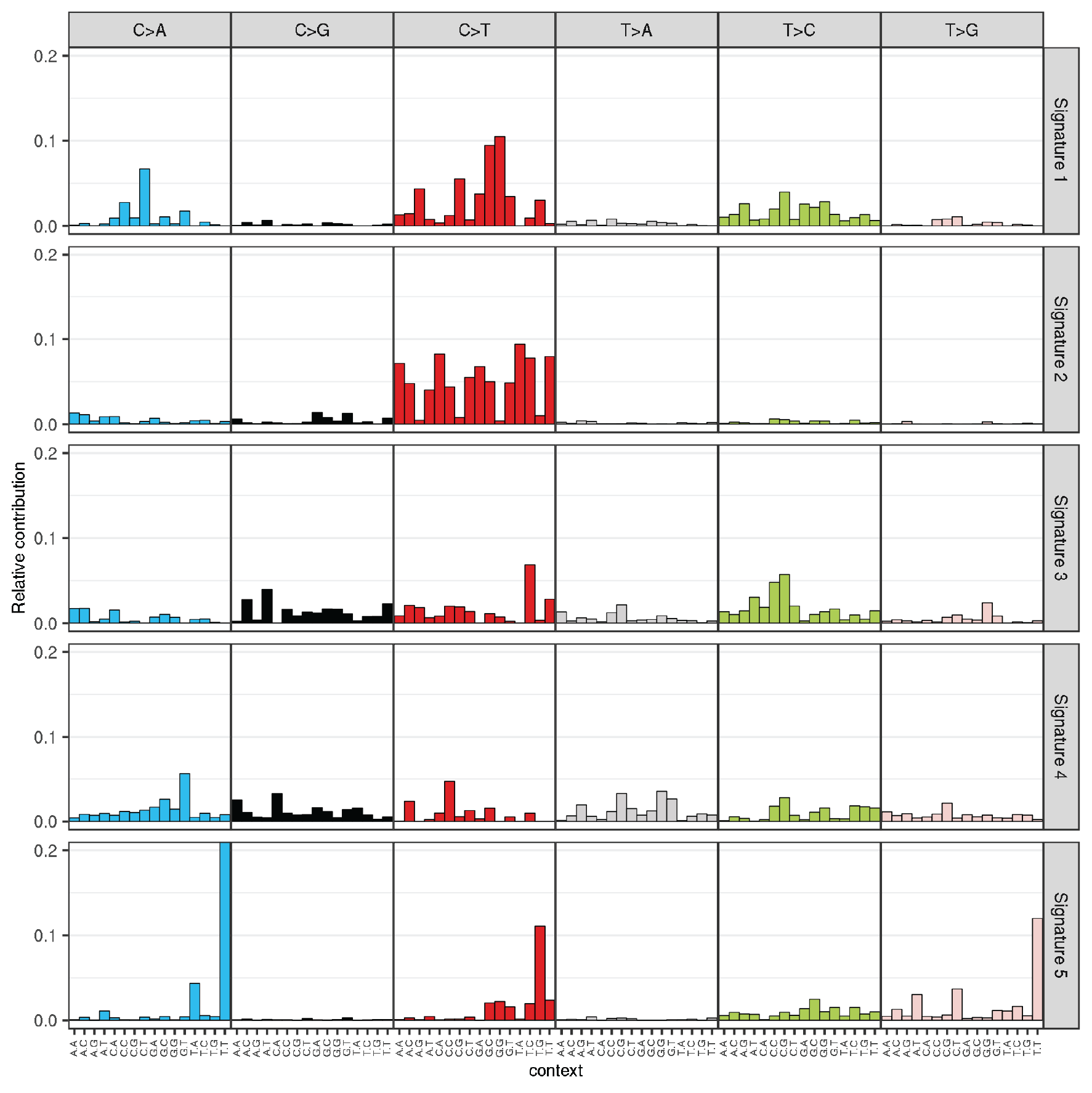
**

Supplementary Figure 3:


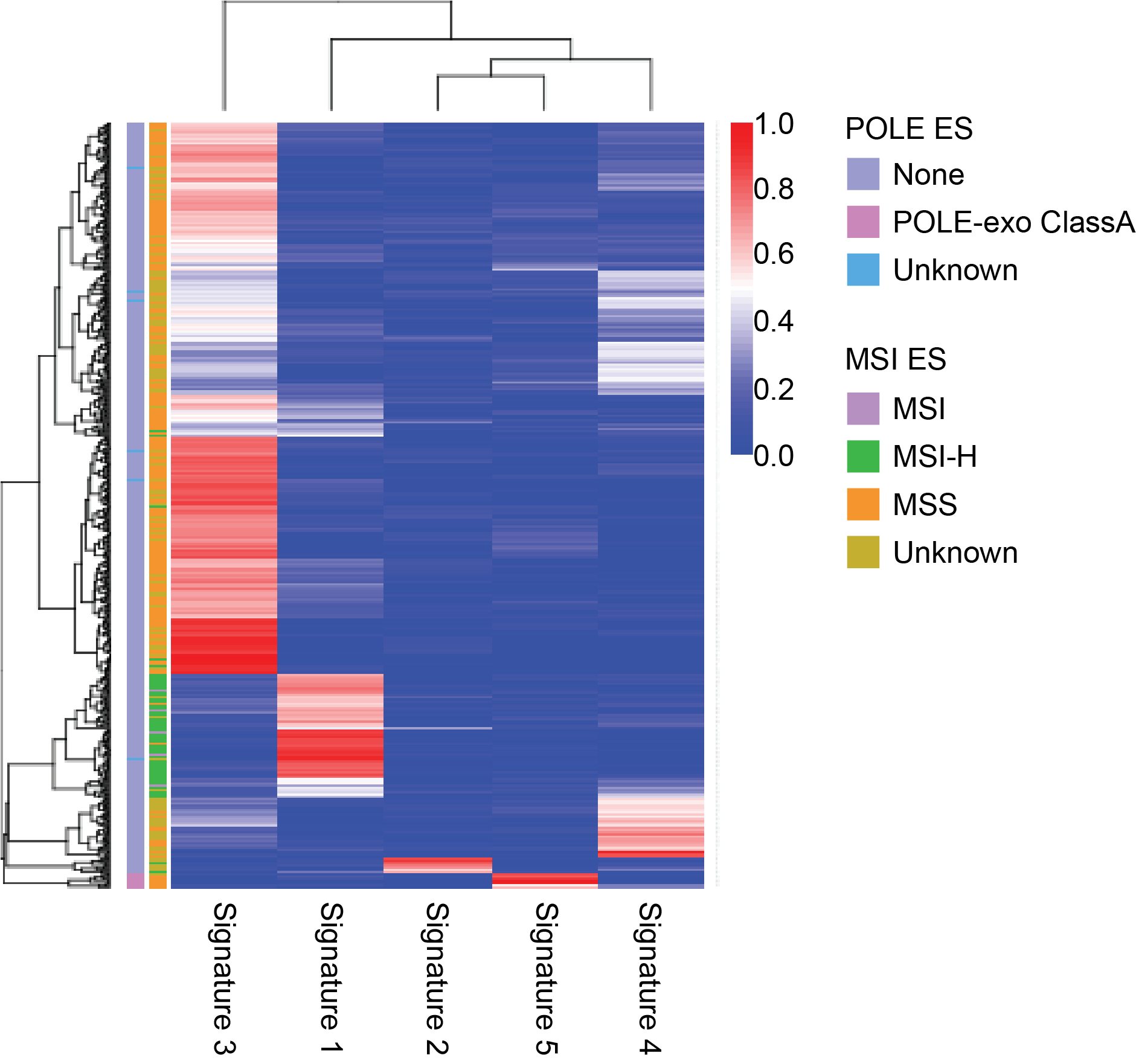


Supplementary Figure 4**:**

**
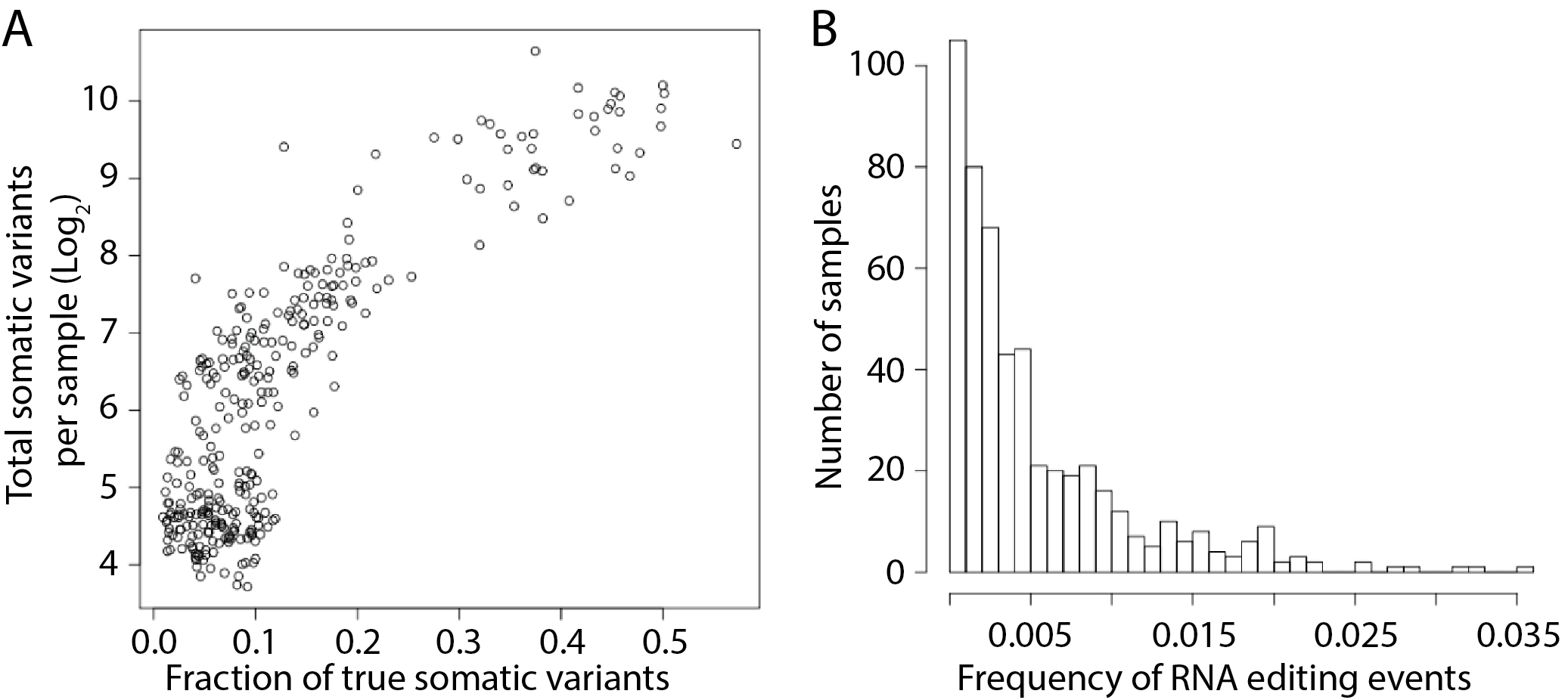
**

Supplement: Supplementary file 1 — Additional file 1: Fig S1: Background regression coefficients of COSMIC signatures in UCEC samples. Histogram of UCEC regression coefficients against (A) a germline signature derived from dbSNP variants, (B) natural aging signature (COSMIC signature 5), (C) MSI-H signature (COSMIC signature 6), and (D) POLɛ signature (COSMIC signature 10). Figure S2: Unsupervised mutational signatures. The mutational frequencies of all five unsupervised signatures identified from UCEC samples and output by mutationalPatterns R package. Figure S3: Sample correlations to unsupervised identified signatures. Sample mutational frequencies were correlated to the top five signatures output by the unsupervised method. The samples are color coded (y-axis bars) based on the clinical annotation of the tumor in regares to MSI and POLɛ status. [file 12920_2021_898_MOESM1_ESM.docx]
